# v-SNARE function in chromaffin cells

**DOI:** 10.1007/s00424-017-2066-z

**Published:** 2017-09-08

**Authors:** Madhurima Dhara, Ralf Mohrmann, Dieter Bruns

**Affiliations:** 1 Molecular Neurophysiology, CIPMM, Medical Faculty, Saarland University, 66421 Homburg/Saar, Germany; 2 Zentrum für Human- und Molekularbiologie, Saarland University, 66421 Homburg/Saar, Germany

**Keywords:** SNARE proteins, Exocytosis, Membrane fusion, SNARE regulators, Ca^2+^-triggered exocytosis

## Abstract

Vesicle fusion is elementary for intracellular trafficking and release of signal molecules, thus providing the basis for diverse forms of intercellular communication like hormonal regulation or synaptic transmission. A detailed characterization of the mechanisms underlying exocytosis is key to understand how the nervous system integrates information and generates appropriate responses to stimuli. The machinery for vesicular release employs common molecular players in different model systems including neuronal and neuroendocrine cells, in particular members of the SNARE (*soluble N-ethylmaleimide-sensitive factor attachment protein receptors*) protein family, *Sec1/Munc18*-like proteins, and other accessory factors. To achieve temporal precision and speed, excitable cells utilize specialized regulatory proteins like synaptotagmin and complexin, whose interplay putatively synchronizes vesicle fusion and enhances stimulus-secretion coupling. In this review, we aim to highlight recent progress and emerging views on the molecular mechanisms, by which constitutively forming SNAREpins are organized in functional, tightly regulated units for synchronized release. Specifically, we will focus on the role of vesicle associated membrane proteins, also referred to as vesicular SNAREs, in fusion and rapid cargo discharge. We will further discuss the functions of SNARE regulators during exocytosis and focus on chromaffin cell as a model system of choice that allows for detailed structure-function analyses and direct measurements of vesicle fusion under precise control of intracellular [Ca]i.

## Introduction

Exocytosis is a fundamental and ubiquitous cellular process, by which secretory organelles merge with the plasma membrane in order to release various cargo molecules (e.g., transmitters, hormones, enzymes, or chemokines). A wealth of experimental evidence has firmly established that members of the SNARE protein family form the minimal molecular machine for membrane fusion [13, 136]. In mammals, the SNARE superfamily contains more than 30 members that exhibit distinct subcellular localization [21, 56] and putatively mediate different intracellular trafficking events [6]. As a common structural feature all SNARE proteins contain a homologous 60–70 amino acid-long domain, also referred to as SNARE motif [144, 150]. SNARE motifs form amphiphatic helices with periodic heptad repeats of hydrophobic residues. The four SNARE helices can assemble into a stable complex [55, 103], as first shown by Rothman and colleagues [127, 128]. In case of exocytosis, the three cognate SNARE proteins synaptobrevin (syb, also known as vesicle associated membrane protein (VAMP)), syntaxin (stx), and SNAP-25 assemble into a functional complex, with syb and stx each contributing one SNARE motif, while SNAP-25 inserts two motifs into the assembly. Although singular SNARE motifs are largely unstructured in solution, the three SNARE motifs of syb, stx, and SNAP-25 spontaneously assemble into a stable complex that is exceptionally resistant to heat denaturation [31] and SDS treatment [49]. As shown in crystallization studies of the Brunger group [138], the four α-helices within the fully assembled SNARE complex form a rod-like coiled-coil bundle. This 12-nm-long core complex is stabilized by nonpolar side-chain interactions of conserved residues in all four helices, thereby forming 15 “hydrophobic layers” numbered from − 7 to + 8 [58, 138]. Intriguingly, a central “0” layer of the complex is composed of conserved polar amino acids: one arginine (R) and three glutamine (Q) residues [32, 159]. This feature is highly conserved throughout the SNARE superfamily [3, 62, 109] and led to the classification of SNAREs into R- and Q-SNAREs, which is used in parallel to the functional categorization in vesicular (v)-SNAREs and target (t)-SNAREs in the context of exocytosis.

While the importance of SNARE proteins for exocytosis has been established beyond any doubt by the action of clostridial neurotoxins (for a detailed review see [105]), by genetic mutants affecting SNARE protein function [20, 33, 157], or by knockout models [8, 12, 26, 121, 129, 130, 148], our knowledge of the exact molecular mechanism of Ca^2+^-regulated exocytosis is still far from being complete. In particular, the SNARE-lipid interactions and mechanistic intermediates leading to membrane merger and fusion pore formation are still elusive. Reduced in vitro models could demonstrate that membrane-anchored SNARE proteins enable fusion of liposomes, albeit at a slow time scale [102, 149]. Yet, the challenge remains to unravel how specific kinetic properties and Ca^2+^ dependency of physiological secretion are conferred to the SNARE machinery. Neuroendocrine cells of the adrenal medulla have been instrumental in elucidating critical mechanistic aspects of Ca^2+^-dependent exocytosis. Here, we will discuss recent advances in our understanding of SNARE function, especially highlighting studies that employed adrenal chromaffin cell as a model system.

## Studying exocytosis in chromaffin cells

Chromaffin cells of the adrenal medulla are specialized neuroendocrine cells belonging to the sympathetic nervous system. Apart from several peptide hormones and bioactive peptides, these cells release either adrenaline (80%) or noradrenaline (20%) from large-dense core vesicles into the bloodstream [36]. Forming a sympathetic paraganglion, chromaffin cells receive excitatory cholinergic innervation from preganglionic splanchnic fibers. Released acetylcholine depolarizes chromaffin cells and elicits action potentials that induce Ca^2+^ influx via voltage-dependent Ca^2+^ channels, which in turn triggers the exocytosis of secretory granules [16, 82, 106] (for detailed review, see [107]). While the specific physiological role of secretion from the adrenal medulla is reviewed in detail in a sister article (Smith and Eiden, this issue), we discuss here the specific properties that make chromaffin cells a superior model system for studying exocytosis.

The greatest benefit of chromaffin cells is given by the accessibility of granule secretion through multiple experimental read-outs: The spherical shape of chromaffin cells makes them well suited for voltage clamping experiments using the patch clamp technique [44]. The compact cellular structure facilitates high-resolution membrane capacitance measurements [99], which can be used to follow exo- and endocytosis based on changes in plasma membrane area. In their seminal work, Neher and Marty observed discrete capacitance steps in the range of 0.4–80 fF using the whole-cell configuration, which is consistent with the fusion of single or multiple vesicles upon stimulation. To induce secretion in membrane capacitance recordings, chromaffin cells can be loaded via the patch pipette with intracellular solutions containing elevated Ca^2+^ levels (nM-μM) [96, 97, 126] or with caged-Ca^2+^ compounds that can be photolyzed by transient UV illumination, granting full control over the intracellular Ca^2+^ concentration [100]. Indeed, Ca^2+^ uncaging experiments in chromaffin cells have been crucial in determining secretion kinetics as well as in delineating vesicle pools with distinct release readiness (the so-called readily releasable pool (RRP) and slowly releasable pool (SRP)), which seem to be associated with different “primed” states of secretory vesicles [145]. The high time resolution of these experiments also allowed to assay the latency of secretion and the Ca^2+^ dependency of release rates, delivering reliable information on ‘stimulation-secretion coupling’ (for detailed review, see [113]). Since chromaffin granules release oxidizable cargo molecules (catecholamines), transmitter discharge from single vesicles can be directly recorded as oxidation currents by a polarized carbon fiber electrode [71]. Such amperometric recordings provide a high temporal resolution, monitoring discrete phases of transmitter discharge from a single chromaffin granule [23, 154]. Apart from electrophysiological methods, chromaffin cells have also been one of the first model systems, in which the fusion of single vesicles with the plasma membrane could actually be visualized. In particular, vesicle movement near the plasma membrane can be easily studied by “total internal fluorescence” microscopy [134, 135]. These experiments exploited the fact that spherical chromaffin cells flatly attach to a glass surface forming a “foot print” and that chromaffin granules can be readily marked and tracked by expression of vesicular cargo (e.g., neuropeptide Y) carrying a fluorophore tag.

The molecular players responsible for biogenesis, targeting, docking, and fusion of secretory vesicles have been well characterized in chromaffin cells [4, 34, 51, 119, 129, 139] and largely match the components in the exocytotic machinery of neurons [14]. This similarity has facilitated the use of chromaffin cells as a model for neuronal exocytosis, covering many mechanistic aspects of synaptic transmission. That being said, chromaffin cells lack morphologically distinct sites for vesicle fusion similar to presynaptic “active zones” in neurons. Still, hot-spots for Ca^2+^ entry and granule secretion have been described in cultured chromaffin cells [95, 117]. Owing to these benefits, it is no surprise that chromaffin cells have been extensively used as the model system of choice for studying proteins, lipids, and second messenger systems controlling magnitude and kinetics of vesicle fusion as well as release rate of cargo molecules from single vesicles. In the following part of this review, we will discuss the mechanisms underlying SNARE-mediated, Ca^2+^-triggered membrane merger, giving special attention to studies that have utilized chromaffin cells to delineate the SNARE action—from priming of vesicles to fusion pore opening and its final expansion.

## The SNARE hypothesis: complex assembly and force transduction

How exactly SNARE complexes induce membrane fusion is key to our understanding of the exocytosis mechanism. In the absence of SNARE proteins, spontaneous membrane fusion rarely occurs, as the negative charges of phospholipid headgroups and their associated hydration shells keep biological membranes at a distance of at least 1–2 nm [77, 115]. Energy-consuming steps like the approximation of membranes against repulsive forces, shedding of the hydration shells, and reorganization of the lipid bilayers put an energetic toll on the fusion reaction, which has been estimated to exceed 40 *κ*_*B*_ *T* (where *κ*_*B*_ is Boltzmann’s constant and *T* is temperature) from theoretical studies [24, 53, 84, 151]. Notably, a recent study utilizing liposome fusion assay approximated the energy cost to be ~ 30 *κ*_*B*_ *T* for membrane merger [35]. While it is widely accepted that formation of the thermodynamically stable SNARE complex delivers the free energy for membrane fusion [73] (for review, see [116]), different energy estimates prevented a definite answer to the question whether the energy of a single SNARE complexes is sufficient to drive membrane merger [73, 80, 81, 152]. However, recent work suggested that one SNARE complex or a low number of SNARE complexes are formed during the fusion of vesicles with the plasma membrane [91, 93, 114, 124, 142].

**Fig. 1:**
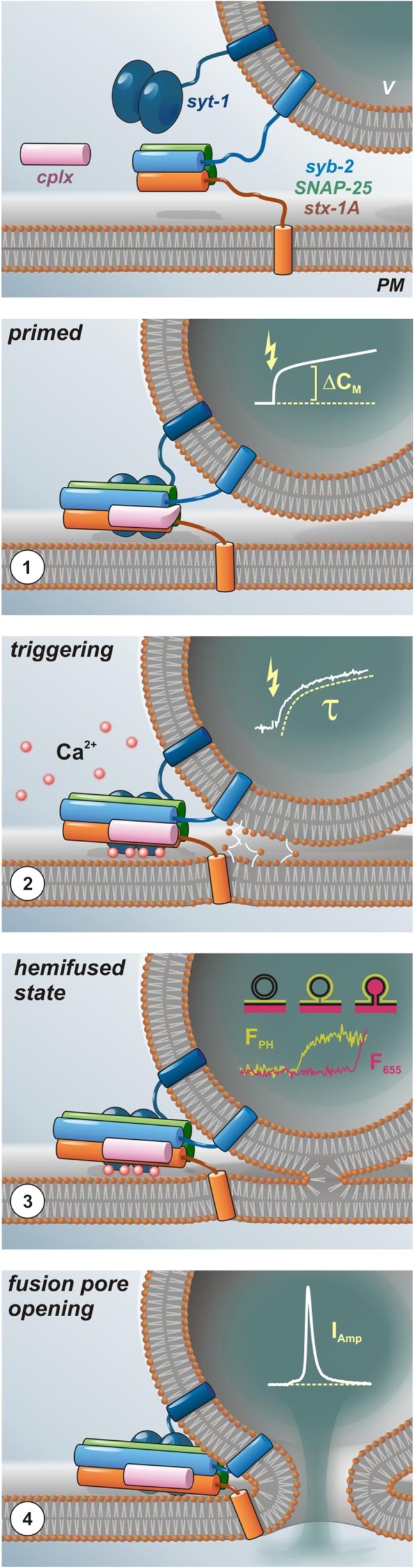
Model of SNARE-mediated Ca^2+^-triggered exocytosis. The core fusion machinery comprises the three SNARE proteins syb-2 (blue), stx-1A (orange; H_abc_ domain not shown), and SNAP-25 (green; depicted without linker for clarity) as well as the accessory factors syt (dark blue) and cplx (pink) (topmost panel). PM, plasma membrane; v, vesicle. (1) *Trans*-SNARE complexes are nucleated during initial tethering and docking steps, and progressive N- to C-terminal zippering of the SNARE complex results in formation of a primed partially assembled intermediate. This primed state is supported by syt and cplx, the latter putatively preventing premature fusion. Primed vesicle pools can be estimated from the size of the initial membrane capacitance increase (inset). (2) Fusion triggering involves Ca^2+^ binding to syt and correlated conformational changes of both, syt and cplx, thereby lifting the fusion clamp and allowing for C-terminal SNARE assembly. Molecular straining of membranes and SNARE TMD dynamics putatively initiate lipid stalk formation. Fusion rate can be estimated by kinetic analysis of membrane capacitance recordings (inset). (3) Merger of the proximal leaflets results in hemifusion, as suggested by imaging of the fluorescently labeled cytosolic leaflet (*F*_PH_) of the plasma membrane versus the fluid phase marker (*F*_655_) in chromaffin cells [163], (kindly provided by L.G. Wu). (4) Rearrangement of the distal leaflets forms the nascent fusion pore, through which cargo molecules diffuse out of the vesicle. Transmitter discharge from single granule, as measured by amperometry (inset), is limited initially by the diameter of the fusion pore neck. TMD conformational properties efficiently regulate the rate of fusion pore expansion, likely by modulating membrane curvature [27]

Based on structural and biochemical data, it has been envisioned that complex assembly starts at the N-termini of the four aligned SNARE motifs and subsequently progresses toward their C-terminal ends [47, 48, 74, 110, 138]. Functional evidence for progressive zippering was delivered by Xu et al. by testing the effect of a monoclonal antibody that recognizes an N-terminal epitope within SNAP-25 (amino acid 20–40) [157]. Characterizing secretion properties in chromaffin cells with membrane capacitance measurements and Ca^2+^ uncaging, they could show that a loose N-terminal SNARE assembly results in a decreased rate of sustained release, suggesting that SNARE complex initialization is hindered. In addition, a selective loss of the fast burst component indicated that the bound antibody somehow delayed the transition of partially zippered to fully assembled complexes, thus determining the balance between SRP and RRP. Another insight into SNARE assembly came from experiments, in which N- or C- terminal protein fragments of the stx-1A SNARE motif were infused in permeabilized PC12 cells. Intriguingly, only a N-terminal but not of a C-terminal fragment inhibited Ca^2+^-triggered release of norepinephrine, which again emphasizes the functional polarization of SNARE motifs [86]. While it would be also possible that SNARE assembly occurs in one step after the Ca^2+^ triggering [143], most evidence points to the occurrence of a distinctive partially assembled fusion intermediate (Fig. 1). Sorensen and colleagues directly tested the idea of progressive SNARE zippering in mouse chromaffin cells and systematically analyzed the phenotypical properties of mutations that interfere with the formation of different hydrophobic SNARE layers [131, 147]. Expressing either SNAP-25 or syb-2 mutants in corresponding null-background chromaffin cells, the Sorensen group performed combined membrane capacitance measurements and Ca^2+^-uncaging experiments in order to analyze vesicle pools and transition kinetics. By this method, they showed that mutations in the membrane proximal C-terminus of the SNARE complex preferentially compromised Ca^2+^-induced fusion triggering, whereas mutations in the middle or in the N-terminus of the complex selectively impeded vesicle priming without detectable changes in triggering rate. These findings support the notion of sequential assembly, starting with zippering at the N-terminus during vesicle priming and ending with C-terminal assembly of the SNARE complex that coincides with membrane fusion.

For regulated exocytosis the SNARE zippering process should be discontinuous allowing for a partially assembled intermediate state, wherein the SNARE complex rests, before Ca^2+^ stimulus induces final membrane fusion. SNARE folding experiments with optical tweezers recently delivered evidence for the existence of such a metastable assembly state [72, 165], which is possibly stabilized by SNARE regulators like complexins [65], as will be discussed in a later chapter. The transition from a partly unstructured, flexible SNARE configuration to a fully assembled rod-like structure should result in mechanical force transfer from the SNARE complex onto the adjoining membrane segments until the relative positions of the transmembrane anchors of stx-1A and syb-2 have converged in the process. Interestingly, an X-ray crystallographic study [133] that analyzed neuronal SNARE complexes containing full-length stx-1A and syb-2 suggested that helical assembly may extend beyond the SNARE motifs, encompassing JMRs and TMDs of both transmembrane proteins. The resulting SNARE force may not only be used to pull the secretory organelle toward the plasma membrane but might also locally strain the lipid bilayers, thereby directly contributing to membrane merger. Based on these considerations, it has been posited that the length of the linker peptide that connects the SNARE motif with the transmembrane domain (TMD) should be critical for fusion efficiency [88]. In case of the v-SNARE syb-2, insertion of a 12 or 24 amino acid-long flexible linker between the SNARE motif and TMD abolished synaptic vesicle exocytosis [25]. Experiments in syb-2/cellubrevin (ceb) double null chromaffin cells expressing syb-2 variants with increasing linker length (insertion of 2–22 amino acids) gradually reduced the primed pool of vesicles, attenuated early fusion pore jitter, and slowed the kinetics of cargo discharge from single vesicle in a linker length-dependent fashion [60]. These results suggested that continuous molecular straining of SNAREs on membranes drives vesicles from priming to membrane merger. A similar requirement for a tight coupling between SNARE motif and TMD has been shown in bovine chromaffin cells [10] and in syb-2^−/−^ hippocampal neurons [43]. In the neuronal system, the “degree” of coupling between syb-2 TMD and SNARE motif affected the synchronized release component of synaptic transmission as well as the waveform of quantal events, again pointing to deficits in vesicle fusion and transmitter discharge. In the same line, insertion of 3 or 7 amino acid-long linkers between the stx-1A TMD and its SNARE motif strongly inhibited evoked synaptic transmission, reiterating the importance of tight mechanical coupling of both elements [164].

## v-SNARE action in protein-lipid interplay and fusion pore opening

The formation of the SNARE complex putatively forces vesicle and plasma membrane into close apposition, which may facilitate the establishment of first hydrophobic contacts, serving as a starting point for significant rearrangements between the merging membranes [149]. In silico analyses have suggested that hydrophobic nucleation events, wherein tails of lipids belonging to opposite membranes interconnect the adjacent leaflets (lipid splay), are highly energy demanding [57, 115, 125]. Therefore, it is possible that the SNARE machinery may not only employ inter-membranous force (by protein-protein interactions) but also direct protein-lipid interactions to facilitate membrane merger (Fig. 1). The juxtamembrane regions (JMR) and TMDs of the SNARE proteins are prime candidates for such SNARE-lipid interactions. Since several interesting ideas about SNARE-lipid recently arose from work with syb-2, we will focus the discussion on v-SNARE-mediated membrane interactions.

### Lipid interactions of the juxtamembrane domain of syb-2

Tryptophan (Trp) residues are frequently found at water-lipid interfaces of transmembrane proteins due to their amphiphatic character [61]. The JMR of syb-2 contains two Trp residues (W89, W90), which are conserved throughout the animal kingdom and could possibly contribute to critical SNARE-lipid interactions during the fusion mechanism. Experiments in reconstitution assays have demonstrated that the two Trp residues in the JMR indeed interact with the phospholipid bilayer [66, 112] and may even define the orientation of the TMD within the vesicular membrane [9]. Interestingly, a site-directed spin-labeling EPR study proposed that substitution of the Trp residues by hydrophilic serine residues accelerated the rate of SNARE complex formation [67], which suggests that JMR-membrane interactions serve an inhibitory role by restricting access to C-terminal portions of the SNARE motif. However, later reports could not confirm this observation, showing that neither kinetics nor efficacy of SNARE complex formation was altered, when Trp residues were mutated [50, 123]. While deletion or substitution of Trps by serine residues did not change SNARE-mediated liposome fusion [123], secretion of human growth hormone in PC12 cells expressing a syb-2 variant containing a W89A/W90A double mutation was substantially reduced [111]. Furthermore, expression of the W89A/W90A double mutant in murine cortical syb-2^−/−^ neurons caused a severe reduction in evoked release and a substantial enhancement of spontaneous release, a phenotype that is reminiscent of the secretion phenotype resulting from the knockdown of the accessory protein complexin (cplx) [87]. Although the W89A/W90A mutation does not alter cplx-SNARE complex interaction, the phenotypic similarities of cplx knockdown and Trp mutations led the authors to conclude that the Trp residues in the JMR are involved in syb-2-cplx interplay [87] or unmask a secondary Ca^2+^ sensor [160] (see also [98]). Fang et al. [30] reported that the W89A/W90A double mutant in v-SNARE-deficient chromaffin cells enhanced the frequency of fusion events upon subtle depolarization of chromaffin cells with 5 mM KCl, increased the quantal size, and reduced secretion in comparison to controls upon stronger stimulation by 100 mM KCl. Contradicting the view of a JMR-based fusion clamp, Borisovska et al. [7] found that substitution of the Trp residues did not change the rate of v-SNARE-dependent exocytosis at submicromolar [Ca]i, but strongly reduced the capacitance increase in response to a stepwise increase in [Ca]I (15–25 μM). Furthermore, no changes in quantal signaling with respect charge, amplitude or kinetics could be detected using carbon fiber amperometry. Additional molecular dynamics simulations of C-terminal syb-2 fragments containing the JMR and the membrane-embedded TMD rather suggested that Trp residues within the JMR of syb-2 play a crucial role in positioning the neighboring lysine and arginine residues at the membrane-water interface, which creates a positive electrostatic potential at the site of action. This positive membrane surface potential could reduce repulsive forces between the negatively charged vesicle and plasma membrane, thereby stabilizing the primed state. Regardless of the exact molecular mechanism, the available data indicate that protein-lipid interplay between the syb-2 JMR and vesicular membrane are important for the maintenance of the primed, release-ready state of the vesicle, either by energetically stabilizing it or by preventing premature fusion.

### Function of the transmembrane domain of syb-2

Previous in vitro analyses suggested that SNARE TMDs in isolation (in the absence of the cytoplasmic SNARE domain) actively promote liposome fusion [69, 70]. Furthermore, experiments in reduced model systems have shown that replacing the SNARE TMDs with lipidic anchors is detrimental for fusion between liposomes [89], cell-cell fusion with ectopically expressed “flipped” SNAREs [41], and lipid nanodisc fusion with liposome [5, 122] and for yeast vacuole fusion [42, 108, 118]. Evidently, SNARE TMDs may actively contribute to membrane merger rather than serving as passive membrane anchors. In sharp contrast, the role of SNARE TMDs in neurotransmitter release from live cells has been controversially discussed. The substitution of the syb-2 TMD with an acylated, lipid-anchoring motif of CSP-α was reported to allow for an substantial rescue of evoked synaptic transmission in murine cortical neurons and also supported spontaneous release of miniature currents similar to wild-type protein [164]. However, the same syb-2/CSP-α chimera was shown to be inefficient in supporting exocytosis in neurons as well as mouse chromaffin cells [17]. Moreover, high-resolution membrane capacitance measurements and carbon fiber amperometry have indicated that CSP-α anchored syb-2 proteins failed to promote efficient exocytosis as well as transmitter discharge from chromaffin granules [27]. These results provided strong evidence for an important role of SNARE TMDs in Ca^2+^-triggered release.

Generally, the fusion pore has been conceived as a purely lipidic structure that results from progressive membrane rearrangements starting with the establishment of a hemifused intermediate (for detailed review, see [68]), Fig. 1). The existence of such a hemifused state, in which only the outer leaflets of two bilayers merge, while the inner leaflets remain intact, was first demonstrated for membrane fusion of influenza hemagglutinin viruses. In this viral system, mutant hemagglutinin without TMD arrested fusion at an intermediated state allowing for lipid mixing but not for content exchange [59, 90]. Later, hemifused intermediates were also shown in SNARE-dependent liposome fusion [158, 161], in cell-cell fusion [41], in lipid nanodisc-liposome fusion [122], and even in Ca^2+^-triggered neurotransmitter release from chromaffin cells [163]. To actively support membrane fusion in this scenario, TMDs-lipid interactions may either facilitate the formation of the initial hemifused state or support the transition to the opened fusion pore. While several recent studies have presented clues, suggesting that v-SNARE TMDs indeed serve important functions in these particular steps, little consent about the underlying mechanism has been reached. Liposome fusion experiments and structural analysis with EPR have pointed out that lipid interactions may switch the conformation of v-SNARE TMD dimers from an open to a closed scissor-like configuration, which could help to modify membrane curvature and thus facilitate fusion [140]. Moreover, results from a fusion assay between v-SNARE-containing lipid nanodiscs and t-SNARE liposomes suggested that a critical number of native v-SNARE TMDs is required to open and maintain the fusion pore [122], implying that the TMD facilitates the transition from lipid stalk to open fusion pore. Extending the idea of v-SNARE-mediated mechanical straining of the membrane, the group of Manfred Lindau investigated the possibility that the SNARE TMDs could be actually pulled through the vesicular membrane and thereby induce reorganization and merger of affected membrane segments. In support of this notion, Ngatchou et al. have shown that addition of polar residues (lysine or glutamate) at the luminal end of syb-2 strongly suppressed exocytosis [101]. Since the inhibition of granule fusion was clearly determined by the polarity of the replacing amino acids, this observation might indicate that this TMD region is shifting its position into the membrane or even transit through it [101]. However, a lot of molecular details with respect to the fate of the syb-2 TMD are still unclear. We performed a systematic structure-function analysis in chromaffin cells and neurons to provide evidence for yet another role of the syb-2 TMD that is tied to the high incidence of helix-destabilizing ß-branched amino acids (isoleucine/valine) within its sequence [27]. Intriguingly, our data showed that substitution of the native TMD core by a leucine-containing peptide that is strictly helical significantly reduced Ca^2+^-triggered release and also slowed down the kinetics of catecholamine discharge from single granules in chromaffin cells. In reverse, TMDs mutants containing an increased number of ß-branched amino acids could again fully support secretion but accelerated the rate of fusion pore expansion over that in wild-type controls [27]. Comparing these functional results with conformational data from molecular dynamic simulations of membrane-embedded syb-2 TMD mutants, we found a direct correlation between the conformational flexibility of the TMD and its ability to facilitate pore expansion kinetics. Overall, these data have suggested that structural flexibility of v-SNARE TMDs might play a role at different steps of the fusion mechanism (Fig. 1). In the initial phase of membrane merger, structural properties of the TMD might promote lipid splay in the neighboring membrane area [115] and thereby facilitate fusion. Similarly, conformational dynamics of the TMD seem to facilitate of fusion pore expansion, most likely by affecting the negative curvature of the narrow fusion pore neck.

Still, alternative hypotheses on fusion pore structure have been brought forward in the literature. For example, SNARE TMDs might be organized in a barrel-like arrangement to directly form a proteinaceous fusion pore that in principle would resemble an ion channel [54, 75, 76]. The idea of a channel-like fusion pore first arose from membrane capacitance measurements in mast cells, where the initial fusion pore conductance was similar to that of a large ion channel, and fusion pores showed a “flickering” behavior just like ion channels [94, 132]. More direct support of this view was then provided by a systematic mutational analysis, in which single amino acids in the stx TMD were substituted by bulky residues, in particular Trp [46]. Performing amperometric recordings in PC12 cells, the authors demonstrated that mutations reducing pore conductance resided on the same face of the TMD helix, thus putatively lining the pore. This analysis was recently also extended to the syb-2 TMD and identified not one but two mutation-sensitive surfaces on the TMD pointing in opposite directions, which would suggest a deviating structural organization of the hemi-channel formed by syb-2 TMDs compared to its counterpart on the plasma membrane [19]. These results have been interpreted as evidence for a fusion pore made up from multiple SNARE TMDs organized in a gap junction-like arrangement in SNARE-bridged membranes [18, 45, 141]. However, the hypothesis of a proteinaceous fusion is difficult to reconcile with the observation that membrane-anchored syb-2 lacking the TMD was still able to support some chromaffin granule exocytosis above the level of v-SNARE null background [17, 27].

In this context, it is important to note that the lipid composition of the adjoining membranes is also a critical determinant of membrane fusion. Previous studies have provided evidence that various species of membrane phospholipids can affect bilayer merger by virtue of their shape or charge (for a detailed review, see [2]). Since putative fusion intermediates require phospholipids to arrange in membrane structures with high curvature [22], it stands to reason that lipids that can support the curvature of the fusion pore neck (negatively curved cytosolic side and positively curved luminal side) also promote fusion. Several studies have demonstrated that manipulation of the lipid composition in neuroendocrine cells alters granule secretion [1, 162]. In particular, application of the curvature perturbing lysophosphatidyl choline (LPC), which induces positive membrane curvature due to its large head-group area relative to the carbonyl chain, has been demonstrated to either reduce or enhance exocytosis depending on the side of action (intra- or extracellularly). In reverse, phospholipids with a negative curvature like oleic acid exerted opposite effects. Still, the challenge remains to elucidate the functional interplay between protein-lipid interfaces and lipid microdomains in exocytosis research.

## Regulatory brakes on SNARE assembly en route to membrane fusion

### Importance of release-ready vesicles

Since SNARE proteins are constitutively active in membrane fusion [149], the fusion process requires additional regulatory factors that allow the exquisite kinetic control and reliability, which are hallmarks of Ca^2+^-regulated exocytosis. As a result, secretory organelles transit through different mechanistic steps in the fusion process, from “docking” (attachment of the vesicles to the plasma membrane) to priming (molecular maturation that render vesicles release competent), and finally Ca^2+^-dependent triggering [155]. As mentioned above, advanced biophysical analyses of single SNARE assembly have shown that cognate v- and t-SNAREs have the propensity to zipper in a discontinuous fashion allowing for metastable intermediates [37, 165]. In the last decade, significant efforts have been made to identify potential SNARE-interacting proteins that may stabilize a fusion intermediate and thus could function as a “fusion clamp” impeding premature fusion. While a detailed review of SNARE regulatory proteins and their mechanistic functions in determining release-ready vesicle pools is beyond the scope of this review, we still want to briefly outline the role of cplx and synaptotagmin (syt), two accessory proteins, whose interplay seems to be intimately tied to establishment and relief of the fusion clamp (Fig. 1). These factors are believed to act in tandem to establish a state of vesicle maturation, from which secretion can progress in a highly synchronized fashion (for detailed review, see [92]).

### Actions of Cplx and Syt in imparting synchronicity to the release apparatus

Genetic deletion of cplx-2 in chromaffin cells strongly diminishes the exocytotic burst (EB) in chromaffin cells, which is usually observed in response to a stepwise increase in intracellular [Ca]i [15, 28]. Similarly, a loss of syt-1 nearly abolishes the readily releasable pool (RRP) component of the EB [146] and slows down the kinetics of the secretion from the slowly releasable pool (SRP) [28]. Furthermore, studies analyzing synaptic transmission at the neuromuscular junction of *D. melanogaster* and *C. elegans* cplx-null mutants as well as work on secretion in cplx-2 knockout chromaffin cells reported a dramatic increase in spontaneous or tonic release, which implies an additional inhibitory effects of cplx on neurotransmission [28, 52, 85]. A similar increase in spontaneous release has also been seen with cplx-1/-2 double knockdown of murine cortical neurons [87]. In contrast, genetic ablation of the putative calcium sensor syt-1 elevated the rate of spontaneous fusion in some [11, 29, 63, 78, 104, 137, 156] but not in other systems [28, 38, 79]. These disparate results may in part be due to substituting syt isoforms, improperly replacing the Ca^2+^ sensor [63, 137] or to secondary effects of the syt-1 knockout phenotype impairing also the inhibitory impact of GABAergic interneurons on spontaneous glutamatergic signaling [153]. In any case, these results indicate that cplx and syt do not always have mechanistically overlapping functions in fusion clamping.

In the quest for the particular mechanisms, by which cplx clamps release, experiments in reduced systems demonstrated that cplx can inhibit fusion between liposomes carrying only v- and t-SNAREs [120] or between cells expressing such flipped SNARE variants [39]. In close correlation, experiments in mouse chromaffin cells showed that the clamp action of cplx-2 persisted even in the common absence of syt-1 and syt-7—the two major Ca^2+^ sensors present in chromaffin cells [28]. Therefore, both in vitro and in vivo experiments support a model, wherein cplx directly hinders SNARE activity, arresting premature vesicle fusion.

If cplx acts as a “prefusion clamp” that stabilizes half-zippered SNARE complexes, it is mandatory that this molecular clamp can be rapidly lifted upon a Ca^2+^ stimulus to enable a synchronous secretory response with short delay. Experimental clues from in vitro studies [40, 64, 83, 120] have suggested that, upon Ca^2+^ binding, syt likely reverses the “clamped” state by affecting the conformation of cplx bound to SNARE complex, which leads to completion of SNARE zippering and membrane merger. Direct evidence for this putative cplx/syt antagonism recently came from experiments in mouse chromaffin cells [28]. Removal of syt-1 in chromaffin cells resulted in slower expansion of the initial narrow fusion pore, as analyzed by carbon fiber amperometry, while additional loss of cplx-2 (cplx-2^−/−^; syt-1^−/−^) reversed this fusion pore phenotype. Furthermore, overexpression of cplx-2 reproduced the syt-1 ko phenotype, indicating competitive actions of both SNARE regulators. Moreover, in the presence of syt-1, cplx-2 deficiency had no effect on the fusion pore expansion rate at high [Ca]i, but increasingly sped up the rate of pore expansion with lowering [Ca]i. These results can be explained well in the context of a syt-1/cplx-2 antagonism, wherein cpx-2 exerts a decelerating (clamping) effect on fusion pore expansion, which is overcome by syt-1 in a Ca^2+^-dependent fashion. Collectively, these results clearly illustrate the antagonistic actions of both SNARE regulators in controlling the kinetics of the early fusion pore.

## Concluding remarks

Based on the available experimental data, it is apparent that SNARE proteins are the major “work horses” driving membrane fusion, while SNARE regulators provide the temporal speed and precision that are essential for fast Ca^2+^-triggered exocytosis. Intriguingly, experimental results from chromaffin cells have pinpointed intermediate steps of membrane fusion (priming, fusion triggering, and slow/rapid pore expansion) that are affected by SNARE force, SNARE TMD-mediated protein-lipid interactions, and SNARE regulators like cplx and syt. Evidently, these results underline the necessity that different molecular players orchestrate and converge on the same cellular process to guide the vesicles from priming to fusion pore opening and its expansion, ensuring rapid cargo discharge with a sub-millisecond precision.

## Abbreviations

SNAREs: *N*-Ethylmaleimide-sensitive factor (NSF) attachment protein receptors
v-SNARE: Vesicular SNARE
t-SNARE: Target SNARE
SNAP25: Synaptosomal-associated protein, 25 kDa
syb-2: Synaptobrevin II
stxIa: Syntaxin Ia
syt: Synaptotagmin
cplx: Complexin
Ceb: Cellubrevin
VAMP: Vesicular associated membrane protein
PC12: Pheochromocytoma cell line
[Ca]i: Intracellular calcium
NMJ: Neuromuscular junction
